# Microbial diversity and abundance in the Xinjiang Luliang long-term water-flooding petroleum reservoir

**DOI:** 10.1002/mbo3.241

**Published:** 2015-02-02

**Authors:** Peike Gao, Huimei Tian, Guoqiang Li, Hongwen Sun, Ting Ma

**Affiliations:** 1 Key Laboratory of Molecular Microbiology and Technology, Ministry of EducationTianjin, 300071, China; 2 College of Life Sciences, Nankai UniversityTianjin, 300071, China; 3 College of Environmental Science and Engineering, Nankai UniversityTianjin, 300071, China

**Keywords:** 16S rRNA, MEOR, microbial community, miseq, QPCR

## Abstract

Microbial populations associated with microbial enhanced oil recovery (MEOR) and their abundance in the Xinjiang Luliang water-flooding petroleum reservoir were investigated using 16S rRNA, nitrate reductases, dissimilatory sulfate reductase, and methyl coenzyme-M reductase-encoded genes to provide ecological information for the potential application of MEOR. 16S rRNA gene miseq sequencing revealed that this reservoir harbored large amounts of taxa, including 155 bacterial and 7 archeal genera. Among them, *Arcobacter*, *Halomonas*, *Marinobacterium*, *Marinobacter*, *Sphingomonas*, *Rhodococcus*, *Pseudomonas*, *Dietzia*, *Ochrobactrum*, *Hyphomonas*, *Acinetobacter,* and *Shewanella* were dominant, and have the potential to grow using hydrocarbons as carbon sources. Metabolic gene clone libraries indicated that the nitrate-reducing bacteria (NRB) mainly belonged to *Pseudomonas*, *Azospirillum*, *Bradyrhizobium*, *Thauera*, *Magnetospirillum*, *Sinorhizobium*, *Azoarcus*, and *Rhodobacter*; the sulfate-reducing bacteria (SRB) were *Desulfarculus*, *Desulfomonile*, *Desulfosarcina*, *Desulfotignum*, *Desulfacinum*, *Desulfatibacillum*, *Desulfatibacillum*, *Desulfomicrobium*, and *Desulfovibrio*; while the methanogens were archaea and belonged to *Methanomethylovorans*, *Methanosaeta*, *Methanococcus*, *Methanolobus*, and *Methanobacterium*. Real-time quantitative PCR analysis indicated that the number of bacterial 16S rRNA reached 10^6^ copies/mL, while the metabolic genes of NRB, SRB, and methanogens reached 10^4^ copies/mL. These results show that the Luliang reservoir has abundant microbial populations associated with oil recovery, suggesting that the reservoir has potential for MEOR.

## Introduction

With an increasing global energy demand and the depletion of oil reserves, water- and chemical-flooding and microbial enhanced oil recovery (MEOR) are currently studied intensively (Youssef et al. 2009; Wackett 2012). In particular, MEOR is considered to be the most economically feasible because of its low energy consumption, low environmental impact, and cost-effectiveness (Youssef et al. 2009; Simpson et al. 2011). This technique uses reservoir microorganisms and their metabolites to reduce crude oil viscosity, enhance permeability of reservoirs, and selectively plug large pore paths to improve oil recovery.

Complex ecosystems comprising various types of microorganisms are present in petroleum reservoirs. Since Bastin et al. 1926 first isolated sulfate-reducing bacteria (SRB) from production water in 1926, culture-dependent and -independent methodologies, in particular, 16S rRNA-based molecular identification methods, have revealed diverse microorganisms inhabiting petroleum reservoirs (Kumaraswamy et al. 2011; Al-Bahry et al. 2013; Lenchi et al. 2013; Okoro et al. 2014). Among them, hydrocarbon-degrading bacteria (HDB), nitrate-reducing bacteria (NRB), SRB, and methanogens are the important populations of reservoir ecosystems, and have critical roles in the microbial enhancing of the oil recovery process (Youssef et al. 2009). The majority of HDB can produce biosurfactants when growing with hydrocarbon as the carbon source. These biosurfactants improve oil emulsification and lower interfacial tension between the oil and water phase, which further improves oil fluidity in oil-bearing reservoirs. Reducing interfacial tensions and decreasing oil viscosity are important mechanisms involved in MEOR. NRB and SRB are common inhabitants of the oil field ecosystem. The increase of H_2_S (production of SRB) is associated with the corrosion of pipelines, platform structures, and other equipment; increases refining costs of oil and gas; plugs reservoirs by the accumulation of sulfides minerals; and increases health risks because of the toxicity of H_2_S. Recently, the stimulation of NRB by the addition of nitrate, nitrite, or nitrate/molybdate mixtures has been used to inhibit SRB propagation by out-competing the growth of SRB (Bodtker et al. 2008; Gao et al. 2014). As the terminal process of the microbial metabolism chain, methanogens reflect the ecological integrality and metabolic activity of a reservoir ecosystem. In addition, methanogens metabolize hydrogen and CO_2_, acetate, methylamines, and dimethylsulfides with the concurrent production of methane that increases reservoir pressure and decreases oil viscosity.

In this study, 16S rRNA gene miseq sequencing, nitrate reductase, dissimilatory sulfite reductase, and methyl coenzyme-M reductase-encoded gene (*napA*, *dsrB*, and *mcrA*) clone libraries were performed to investigate the microbial communities and the distribution of NRB, SRB, and methanogens in the Luliang water-flooding petroleum reservoir in the XinJiang Oilfield. This reservoir will improve oil recovery by stimulating reservoir microorganisms. Therefore, the primary objective of the study was to provide ecological information on microbial populations and the biological control potential for SRB. This study also provides us the opportunity to evaluate the performance of 16S rRNA gene miseq sequencing for the analysis of reservoir microbial community by the metabolic gene clone libraries and gene quantification.

## Materials and Methods

### Sample collection and DNA extraction

**Figure 1:**
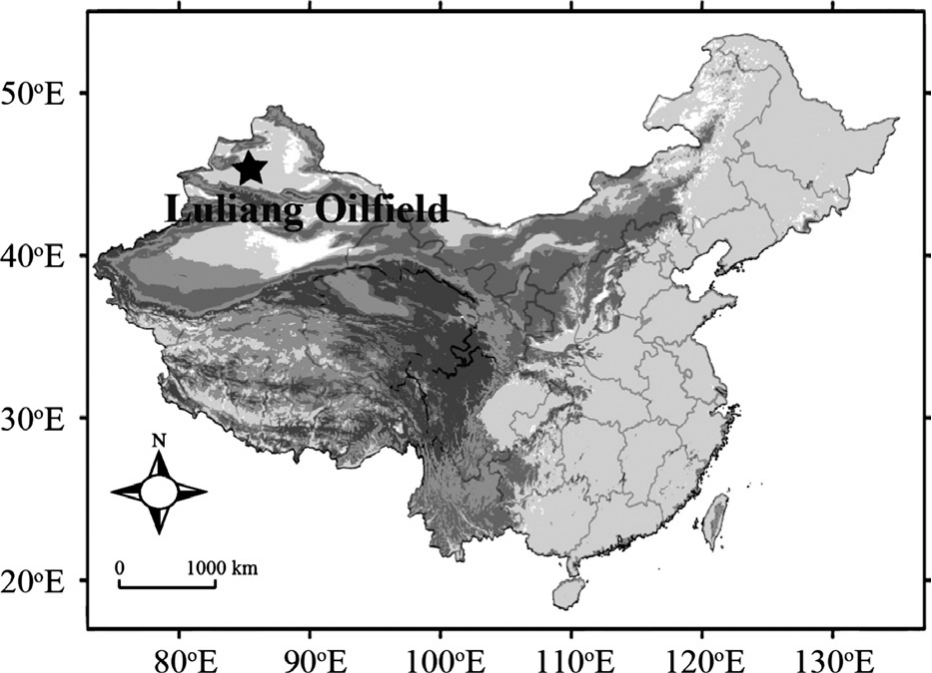
The geographical position of Luliang oilfield that is located in northwest of China. In this oilfield, injected water samples (Lu3064 and Lu3084) and produced water samples (Lu3065 and Lu3096) were collected on October 2012.

Samples of injected water and produced water (oil-water mixture) were collected from the wellhead of injection and the production wells of a mesothermic water-flooding reservoir located in the Xinjiang Luliang Oil Field by PetroChina field personnel (Fig.1). This field block is a homogeneous sandstone reservoir with an average permeability of 522 × 10^−3 ^*μ*m^2^, and has been water-flooded since 2001. The water samples were collected randomly on October 2012, from sampling valves located on the production wellhead. Approximately 25 L of each sample were immediately sealed to avoid contamination and oxygen intrusion. The bottles were then transported to the laboratory as soon as possible for further analysis (7 days later). Microbial cells were collected from a 5 L water sample by centrifugation at 4°C for 15 min at 10,000*g* in a high-speed centrifuge (Beckman, CA 92821, USA). The genomic DNA was extracted as described by Li et al. (2014). To collect as much of the microbial genomes as possible, the collected cells were resuspended with a TE buffer, and then lysed using a mini bead-beater (BioSpec, Bartlesville, OK 74005, USA) at 4°C and 200 rpm for 1 min at room temperature with 0.1 mm glass beads. DNA was extracted from the suspension solution using an AxyPrep™ Bacterial Genomic DNA Miniprep Kit (Axygen Biosciences, Tewksbury, MA 01876, USA) according to the manufacturer's instructions and then stored at −80°C for subsequent study.

### Real-time quantitative PCR analysis of microbial abundance

Evaluation of microbial community abundance by quantitative PCR (QPCR) was performed using 16S rRNA and *napA*, *dsrB*, and *mcrA* genes as molecular markers. Reactions were performed using the FastStart Universal SYBR Green Master PCR mix (Roche Applied Science, Mannheim, Germany) in a Bio-Rad iQ5 Sequence detection system Bio-Rad, CA 92821, USA. QPCR of bacterial 16S rRNA genes were performed with the primer set 8F (5′-AGA GTT TGA T(CT)(AC) TGG CTC-3′) and 338R (5′-GCT GCC TCC CGT AGG AGT-3′) as described by Schulz et al. (2010) and Li et al. (2013). QPCR of *napA*, *dsrB*, and *mcrA* were performed with the primer sets described in the *Clone library construction of napA, dsrB*, *and mcrA genes*. Plasmids containing the target genes were used as standards. The plasmid DNA concentration was determined on a Nanodrop spectrophotometer (Thermo Fisher Scientific, Wilmington, DE). The copy number of the target genes in the initial standard was calculated directly from the concentration of the extracted plasmid DNA. Gene copy numbers in unknown samples were determined based on standard curves constructed from 10-fold serial dilutions of the standard. Amplification efficiencies were calculated from the slope of standard curves. The specificity of PCR amplification was determined using the melting curve.

### Miseq sequencing of partial 16S rRNA genes and sequence analysis

The V4 region of 16S rRNA gene (300–350 bp) was amplified with broadly conserved primer set 515f (GTG CCA GCM GCC GCG GTAA) and 806r (GGA CTA CHV GGG TWT CTA AT). The primer set was reported to be able to yield optimal community clustering with sequences of this length (Caporaso et al. 2011). PCR reactions were performed following the protocol described in Caporaso et al. Amplicon sequencing was conducted on an Illumina MiSeq platform at Novogene co., Beijing, China. Pairs of reads from the original DNA fragments were merged using FLASH (Magoc and Salzberg 2011). Sequences were then analyzed using the QIIME (Caporaso et al. 2010) and UPARSE pipeline (Edgar 2013). First, the reads were filtered using QIIME quality filters with default parameters. Then, a UPARSE pipeline was used to pick operational taxonomic units (OTUs) at 97% similarity. The resulting representative sequence set was aligned and given a taxonomic classification using RDP (Wang et al. 2007). The microbial distribution in the water samples was visualized using R package based on community composition information at taxonomic levels.

### Clone library construction of napA, dsrB, and mcrA genes

Primer set napAf1 (5′-C TGG ACI ATG GGY TTI AAC CA-3′) and napAr1 (5′-CC TTC YTT YTC IAC CCA CAT-3′) were used to amplify *napA* gene (490 bp) (Feng et al. 2011). DSRp2060F (5′-CAA CAT CGT YCA YAC CCA GGG-3′) and DSR4R (5′-GTG TAG CAG TTA CCG CA-3′) were used to amplify *dsrB* gene (390 bp) (Geets et al. 2006). Primer set mcrAF (5′-GGT GGT GTM GGD TTC ACM CAR TA-3′) and mcrAR (5′-CGT TCA TBG CGT AGT TVG GRT AGT-3′) were used for the amplification of *mcrA* genes (450 bp) (Steinberg and Regan 2008). The PCR reaction mixtures and conditions are described in the supporting information. The purified PCR products were cloned into *Escherichia coli* using pEasy-T1 clone vector according to the manufacturer's instructions. The sequences of inserted PCR products were determined with an automated ABI 3730 DNA sequencer using M13 universal sequencing primers.

The retrieved *napA*, *dsrB*, and *mcrA* genes nucleotide sequences were truncated to exclude primers and vector sequences using the FinchTV 1.4.0 program (Wang et al. 2010), and were then translated into protein sequences using the “transeq” algorithm of the EMBOSS program. Deduced protein sequences were compared with sequences in the NCBI Gene Bank database, and were grouped into OTUs based on species taxa. Distance-based evolutionary trees were constructed using the neighbor-joining method with 1000 bootstrap replicates with MEGA 4 (Tamura et al. 2007).

### Sequence accession numbers

The 16S rRNA genes reads were deposited in the National Center for Biotechnology Information (BioProject ID: PRJNA252404, http://www.ncbi.nlm.nih.gov/bioproject/252404). The sequences of *dsr*B genes were deposited in the GenBank database under accession numbers KC466037 to KC466050; the sequences of *nap*A genes were deposited under accession numbers KC466065 to KC466079; and the sequences of *mcr*A genes were deposited under accession numbers KC466051 to KC466064.

## Results

### Physicochemical characteristics of the Luliang reservoir

The Luliang water-flooding reservoir is located in the Xinjiang Oil Field, northwest China. This block has been water-flooded since 2001, with an average water content of 80.3%. The formation temperature is ∽42°C. The average porosity is 29.9%, with an average permeability of 522 × 10^−3^ *μ*m^2^. The physicochemical characteristics of the injected (Lu3064 and Lu3084) and produced (Lu3065 and Lu3096) water samples indicate that the concentrations of sodium, potassium, calcium, magnesium, and manganese are suitable for microbial growth (Table1). The nitrate and phosphate levels were lack for microbial growth. Sulfate (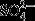) concentrations were between 4.9 and 116.2 mg/L, indicating that sulfate reduction could occur in this reservoir (Bodtker et al. 2008).

**Table 1:** Chemical properties of water samples obtained from Luliang reservoir

Environmental characteristic	Lu3064	Lu3084	Lu3065	Lu3096	Average
Well type	Injection well		Production well		–
Water cut, %	–	–	82%	85%	–
pH	5.5–6.0	5.5–6.0	5.5–6.0	5.5–6.0	5.5–6.0
Salinity	12478	10635	10177	9214	10700
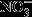 , mg/L	<0.1	<0.1	<0.1	<0.1	<0.1
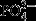 , mg/L	<0.1	<0.1	<0.1	<0.1	<0.1
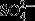 , mg/L	4.9	116.2	14.0	23.1	39.55
Cl^−^, mg/L	6640	5640	5050	4600	5482.5
Na^+^, mg/L	5460	4460	4759	4516	4798.5
K^+^, mg/L	56.8	64.9	44.1	49.3	53.8
Ca^2+^, mg/L	284.7	332.1	281.9	181.6	299.6
Mg^2+^, mg/L	31.6	21.7	27.8	26.03	26.8

Detection limit is 0.1 mg/L.

### Quantification of microbial communities

**Figure 2:**
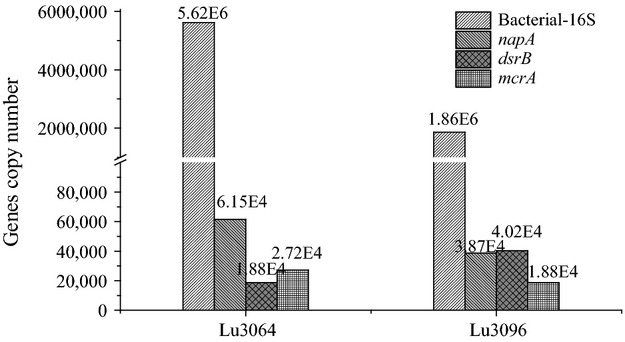
The copy numbers of 16S rRNA, *napA*, *dsrB*, and *mcrA* genes in the injected and produced water samples obtained from Luliang reservoir.

Total bacteria, NRB, SRB, and methanogens were estimated based on the quantification of bacterial 16S rRNA, *napA*, *dsrB*, and *mcrA* genes using QPCR methods. The copy numbers of 16S rRNA, *napA*, *dsrB*, and *mcrA* genes in the injection and production water samples ranged from 1.86 × 10^6^ to 5.62 × 10^6^ copies/mL, 3.87 × 10^4^ to 6.15 × 10^4^ copies/mL, 1.88 × 10^4^ to 4.02 × 10^4^ copies/mL, and 1.88 × 10^4^ to 2.72 × 10^4^ copies/mL, respectively (Fig.2). Assuming that a bacteria contained one copy of the metabolic functional genes (Schulz et al. 2010; Li et al. 2013) and 3.6 copies of 16S rRNA genes per cell genome (Harms et al. 2003), the total bacterial density was calculated as 5.17 × 10^5^ to 1.56 × 10^6^ cells/L. The number of NRB, SRB, and methanogens reached 10^4^ cells/L, which suggests that the ratio of these populations to total bacteria ranged from 39.4‰ to −74.9‰, 12.0‰ to 77.8‰, and 17.4‰ to 36.4‰, respectively.

### Statistical analysis of 16S rRNA miseq sequencing and the metabolic gene clone library

A total of 16,568 to 115,661 high-quality 16S rRNA gene sequences were retrieved from the four injected and produced water samples (Table2). The numbers of OTUs in each injected and produced water sample ranged in size from 1085 to 1515 (Table2). In combination with the community composition and relative abundance, the number of bacterial and archaeal sequences was calculated, with bacterial sequences in each injected and produced water samples ranging in size from 16554 to 115205, whereas only 13–455 archaeal sequences were obtained (Fig. S1). A total of 40–50 metabolic gene sequences were retrieved from the *nap*A, *dsr*B, and *mcr*A gene clone libraries, with 4–11 OTUs per sample (Table2).

**Table 2:** Statistical analysis of 16S rRNA miseq sequencing and metabolic genes clone libraries

Library	Lu3084	Lu3065	Lu3064				Lu3096			
	16S	16S	16S	*napA*	*dsrB*	*mcrA*	16S	*napA*	*dsrB*	*mcrA*
Sequences	16568	19692	115661	45	42	40	48400	40	50	41
OTUs	1238	1197	1515	11	8	7	1085	4	6	7
Shannon	7.07	7.11	6.73	–	–	–	6.70	–	–	–
Coverage, %	99.9	99.9	99.9	88.1	98%	95%	99.8	97.5%	96%	97.6%

OTU, operational taxonomic units.

### Phylogenetic analysis of bacterial 16S rRNA genes

**Figure 3:**
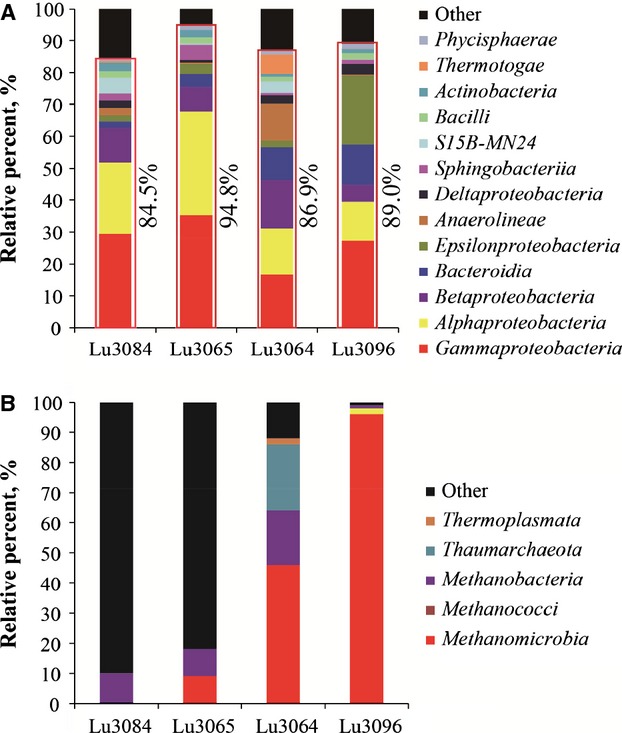
The relative proportion of (A) bacterial and (B) archaeal phylogenetic groups at class level in the injected (Lu3064 and Lu3084) and produced (Lu3065 and Lu3096) water samples obtained from Luliang reservoir.

The classification and phylogenetic analysis indicated that all the bacterial sequences fell within 38 phyla (Fig. S2). The phylum *Proteobacteria*, *Bacteroidetes*, *Chloroflexi*, and *Firmicutes* predominated, representing 77.94–93.89% of the bacterial communities in the water samples (Fig. S2). The remaining bacterial sequences were mainly assigned to the phylum *Cyanobacteria*, *Actinobacteria*, *Thermotogae*, *Planctomycetes*, *Chlamydiae*, *Spirochetes*, *Synergistetes*, and candidate division WPS-2 (Fig. S2). At class level, 84.5–94.8% of bacterial sequences were assigned to *Gammaproteobacteria*, *Alphaproteobacteria*, *Betaproteobacteria*, *Bacteroidia*, *Epsilonproteobacteria*, *Anaerolineae*, *Deltaproteobacteria*, *Sphingobacteriia*, *Bacilli*, *Actinobacteria*, *Thermotogae,* and *Phycisphaerae* (Fig.3). A total of 155 genera were observed, accounting for 42.4–53.5% of the total bacterial communities in the water samples (Table S1).

**Figure 4:**
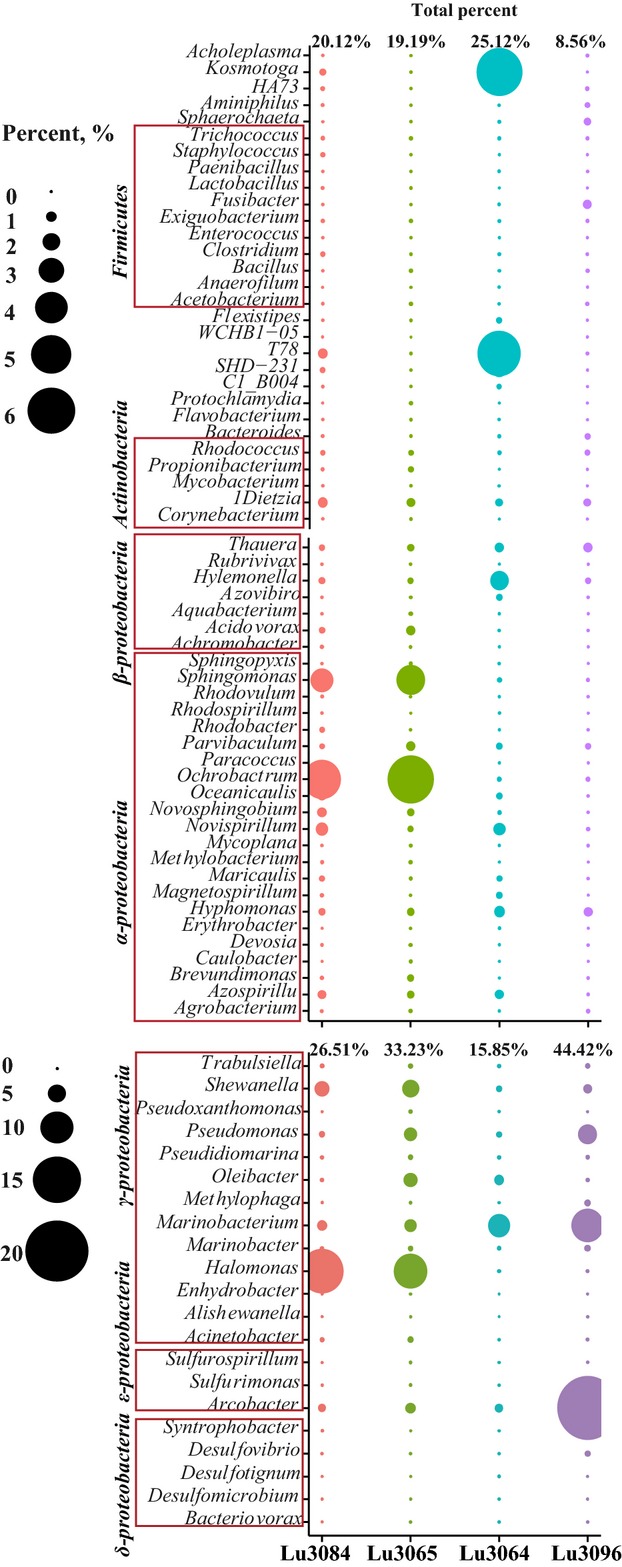
The dominant bacterial genera and their relative abundance in the injected (Lu3064 and Lu3084) and produced (Lu3065 and Lu3096) water samples obtained from Luliang reservoir. These genera were mainly affiliated with the phylum of *Proteobacteria*, *Actinobateria,* and *Firmicutes*.

*Proteobacteria* were mainly detected in this reservoir, with a relative percent of 16.6–35.3% in each water sample. Among them, *Gammaproteobacteria* were most dominant, with a relative percent of 16.6–35.3% in each water sample (Fig.3). Among them, *Marinobacter*, *Pseudomonas*, *Acinetobacter*, *Halomonas*, and *Shewanella* were most numerous (Fig.4). The remaining genera were closely related to *Alishewanella*, *Pseudidiomarina*, *Trabulsiella*, *Enhydrobacter*, *Methylophaga*, and *Pseudoxanthomonas*. *Alphaproteobacteria*, accounting for 16.6–35.3% of each community, were the second most common bacteria in the reservoir with 22 frequently detected genera (Figs.3 and 4). Among them, *Agrobacterium*, *Rhodobacter*, *Rhodospirillum*, and *Azospirillum* were dominant. *Betaproteobacteria* made up 5.3–15.3% of the reservoir bacterial communities (Fig.3). The most frequently sequenced genera were *Achromobacter*, *Acidovorax*, *Aquabacterium*, *Hylemonella*, *Rubrivivax*, *Azovibrio,* and *Thauera* (Fig.4). *Epsilonproteobacteria* accounted for 1.9–21.6% of the bacterial communities in the reservoir (Fig.3), with *Arcobacter*, *Sulfurospirillum,* and *Sulfurimonas* most frequently detected (Fig.4). *Deltaproteobacteria* accounted for 0.87–3.28% of the bacterial communities (Fig.3) and the dominant genera were *Desulfomicrobium* and *Desulfovibrio*, as well as *Syntrophobacterales* (Fig.4). *Actinobacteriae* accounted for 0.96–2.58% of the bacterial communities in the reservoir (Fig.3) and the dominant genera were *Dietzia*, *Rhodococcus*, *Mycobacterium*, *Corynebacterium*, and *Propionibacterium* (Fig.4).

*Bacteroidia* and *Bacilli* were also detected in the reservoir, with relative abundances of 1.89–12.7% and 1.46–2.11%, respectively (Fig.3). In the *Bacteroidia* class, most sequences could not be identified at the genus level with at least 80% confidence. Only genus *Bacteroides* was detected, with a relative abundance of 0.09–0.45% (Fig.4). In the *Bacilli* class, the dominant genera were *Bacillus*, *Paenibacillus*, *Staphylococcus*, *Exiguobacterium*, *Trichococcus*, *Enterococcus,* and *Lactobacillus* (Fig.4).

### Phylogenetic analysis of archaeal 16S rRNA genes

Only 13 and 17 archaeal 16S rRNA gene sequences were obtained from Lu3084 and Lu3065 water samples, while a total of 455 and 389 archaeal sequences were retrieved from Lu3064 and Lu3096 water samples (Fig. S1), respectively. The classification and phylogenetic analysis indicated that all the archaeal sequences retrieved from Lu3064 and Lu3096 water samples fell within the *Crenarchaeota* and *Euryarchaeota* phyla. The classes *Thaumarchaeota*, *Methanobacteria*, *Methanococci,* and *Methanomicrobia* accounted for 86% and 99% of the archaeal communities in Lu3064 and Lu3096 water samples, respectively. The dominant genera were *Methanobacterium*, *Methanothermobacter*, *Methanococcus*, *Methanocalculus*, *Methanomethylovorans*, *Methanolobus,* and *Nitrosopumilus* (Table S3).

### Phylogenetic analysis of napA, dsrB, and mcrA genes

**Figure 5:**
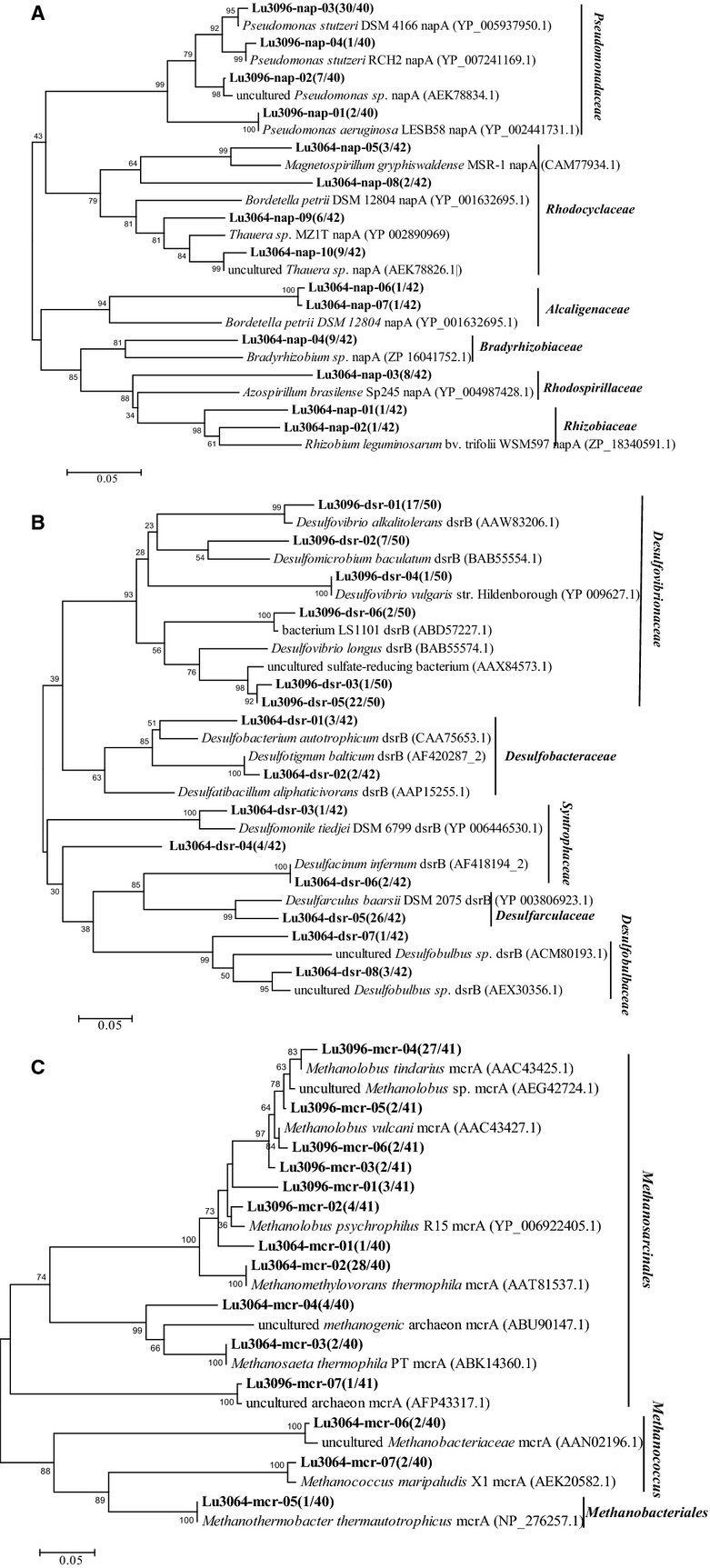
Phylogenetic tree of *napA* (A), *dsrB* (B) and *mcrA* (C) protein sequences detected in the injected and produced water samples obtained from Luliang reservoir. Distance-based evolutionary trees were constructed by the neighboring-joining method with 1000 bootstrap replicates. The scale bar represents 0.05 inferred substitutions per nucleotide position. Percentages of bootstrap support are indicated at the branch points. The nucleotide sequence accession numbers and clones of each OTU were presented in brackets. OTU, operational taxonomic units.

The *napA* sequences retrieved from this reservoir were mainly assigned to *Azospirillum* sp., *Magnetospirillum magnetotacticum*, *Rhodobacter*, *Sphaeroides*, *Bradyrhizobium* sp., *Sinorhizobium fredii*, *Azoarcus* sp., *Thauera* sp., *Candidatus Accumulibacter*, *phosphatis clade* IIA, *Bordetella petrii*, *Laribacter hongkongensis*, *Pseudomonas stutzeri,* and *Pseudomonas aeruginosa* (Table3 and Fig.5A). The retrieved *dsrB* sequences were assigned to *Desulfarculus baarsii*, *Desulfomonile tiedjei*, *Desulfacinum infernum*, *Desulfosarcina* sp., *Desulfobulbus* sp., *Desulfotignum balticum*, *Desulfatibacillum alkenivorans*, *Desulfovibrio alkalitolerans*, *Desulfovibrio aminophilus*, and *Desulfovibrio vulgaris* (Table3 and Fig.5B). The retrieved *mcrA* sequences were assigned to *Methanococcus maripaludis*, *Methanococcus vannielii*, *Methanothermococcus thermolithotrophicus*, *Methanosaeta thermophila*, *Methanolobus psychrophilus*, *Methanolobus tindarius*, *Methanolobus vulcani*, uncultured *Methanomethylovorans* sp., uncultured *Methanosarcinales* archaeon, *Methanobacterium formicicum,* and *Methanobacterium thermoautotrophicum* (Table3 and Fig.5C).

**Table 3:** NRB, SRB, and methanogens detected in metabolic genes clone libraries

Metabolic function	Order	Family	Closest species (obtained from NCBI)
Nitrate-reducing bacteria	*Rhodospirillales*	*Rhodospirillaceae*	*Azospirillum* sp. B510
			*Magnetospirillum magnetotacticum*
	*Rhodobacterales*	*Rhodobacteraceae*	*Rhodobacter sphaeroides f*. sp
	*Rhizobiales*	*Bradyrhizobiaceae*	*Bradyrhizobium* sp.
		*Rhizobiaceae*	1*Sinorhizobium fredii*
	*Rhodocyclales*	*Rhodocyclaceae*	1*Azoarcus* sp. KH32C
			*Thauera* sp. MZ1T
			1*Candidatus Accumulibacter*
			1*phosphatis* clade IIA
	*Burkholderiales*	*Alcaligenaceae*	1*Bordetella petrii*
	*Neisseriales*	*Neisseriaceae*	1*Laribacter hongkongensis*
	*Pseudomonadales*	*Pseudomonadaceae*	*Pseudomonas stutzeri*
			*Pseudomonas aeruginosa*
Sulfate-reducing bacteria	*Desulfarculales*	*Desulfarculaceae*	1*Desulfarculus baarsii*
	*Syntrophobacterales*	*Syntrophaceae*	1*Desulfomonile tiedjei*
			1*Desulfacinum infernum*
	*Desulfobacterales*	*Desulfobacteraceae*	1*Desulfosarcina* sp
		*Desulfobulbaceae*	Uncultured *Desulfobulbus* sp.
			*Desulfotignum balticum*
		*Desulfobacteraceae*	1*Desulfatibacillum alkenivorans*
	*Desulfovibrionales*	*Desulfovibrionaceae*	*Desulfovibrio alkalitolerans*
			*Desulfovibrio aminophilus*
			*Desulfovibrio vulgaris* RCH1
Methanogens	*Methanococcales*	*Methanococcaceae*	*Methanococcus maripaludis*
			*Methanococcus vannielii*
			1*Methanothermococcus thermolithotrophicus*
	*Methanosarcinales*	*Methanosaetaceae*	1*Methanosaeta thermophila*
		*Methanosarcinaceae*	*Methanolobus psychrophilus*
			*Methanolubus tindarius*
			*Methanolobus vulcani*
			*Methanomethylovorans* sp.
			*Methanosarcinales* archaeon
	*Methanobacteriales*	*Methanobacteriaceae*	*Methanobacterium formicicum*
			*Methanobacterium thermoautotrophicum*
			*Methanobacterium* sp. SA-12

1 Represents microbial populations that only detected by metabolic genes clone libraries. The remaining microbial populations were detected by both of metabolic genes clone libraries and 16S rRNA gene miseq sequencing.

## Discussion

We investigated the microbial communities and the distribution of NRB, SRB, and methanogens in the Luliang water-flooding petroleum reservoir in the XinJiang Oil Field. The results provide ecological information on the microbial composition and the biological control potential for SRB during the stimulation of reservoir microorganisms to enhance oil recovery. The results indicate that the reservoir harbors diverse microbial populations. Based on 16s rRNA miseq sequencing, a total of 38 bacterial phyla and 155 genera were observed in the reservoir. In contrast with previous research (Tang et al. 2012; Lenchi et al. 2013; Okoro et al. 2014), this is the first study to show that so many microbial populations inhabit an oil reservoir. Statistical analysis of the 16S rRNA miseq sequencing indicated that 16,568 to 115,661 high-quality 16S rRNA gene sequences were retrieved from the water samples. The sequencing depth was ∽3- to 20-fold for 454 pyrosequences (assuming 5000 sequences per library), whereas it was 50–400 fold for the 16S rRNA gene clone library (assuming 300 clone per library). Miseq sequencing provides an opportunity to investigate the microbial community with an unprecedented level of detail. However, the current sequencing depth is still limited, in particular, for methanogens. In the present study, the bacterial and archaeal V4 region of 16S rRNA gene was simultaneously amplified with primer set 515f and 806r for miseq sequencing. Up to 16,554–115,205 bacterial sequences were obtained per sample, whereas only 13–455 archaeal sequences were obtained. The QPCR analysis indicated that the NRB, SRB, and methanogens only accounted for 39.4–74.9‰, 12.0–77.8‰, and 17.4–36.4‰ of the total bacteria, respectively. Therefore, in theory, only 12–77.8 NRB, SRB, or methanogens could be detected in the 16S rRNA gene library with 1000 sequences, suggesting the need for deeper sequencing for the detection of rare microbial species. This illustrates that even when water samples were obtained from the same production well (M17-10) at the same time and with the same sampling method, SRB were only detected in the Tang et al. (2012), but not by Wang et al. (2012).

Based on the RDP's FunGene library (Table S2) (http://fungene.cme.msu.edu//index.spr) (Cole et al. 2009), the bacterial populations with alkane monooxygenase gene (*alk*) were collected and categorized. In *Gammaproteobacteria*, *Marinobacter*, *Pseudomonas*, *Acinetobacter*, *Halomonas*, and *Shewanella* dominated, and have been reported to be able to degrade hydrocarbons. Furthermore, *Marinobacterium*, *Pseudomonas*, and *Acinetobacter* have also been described as halophilic oil-utilizing and rhamnolipids-producing bacteria (Abdel-Mawgoud et al. 2010; Satpute et al. 2010). In *Alphaproteobacteria*, the abundant species of *Brevundimonas*, *Ochrobactrum*, *Hyphomonas*, *Paracoccus*, and *Sphingomonas* have been reported to be able to degrade hydrocarbons. Among *Betaproteobacteria*, *Thauera* was reported to be able to reduce nitrate as well as degrade aromatic compounds (Song et al. 2000; Tang et al. 2012). Among *Actinobacteriae*, the dominant genera of *Dietzia*, *Rhodococcus,* and *Mycobacterium* are able to degrade hydrocarbons or produce biosurfactants (Cole et al. 2009; Wang et al. 2011; Xia et al. 2011). These microorganisms and the produced biosurfactants play important roles in enhancing oil recovery.

*Alphaproteobacteria* were the second most common bacteria in this reservoir. Among them, *Agrobacterium*, *Rhodobacter*, *Rhodospirillum*, and *Azospirillum* are closely related to denitrification. Within the *Betaproteobacteria*, *Thauera* has been reported to be able to reduce nitrate and degrade aromatic compounds (Song et al. 2000; Tang et al. 2012). Among *Epsilonproteobacteria*, most *Sulfurospirillum* and *Arcobacter* are associated with the cycling, oxidization, and reduction of sulfur and nitrogen (Tang et al. 2012). The dominant *Deltaproteobacteria* were affiliated with iron and sulfate reducers of *Desulfomicrobium* and *Desulfovibrio*, and with *syntrophic* bacteria of the *Syntrophobacterales* (Fig.4). The syntrophic bacteria of order *Syntrophobacterales* may convert propionate and butyrate to methanogenic substrates (Schmidt et al. 2014). The dominant methanogens were *Methanobacterium*, *Methanothermobacter*, *Methanococcus*, *Methanocalculus*, *Methanomethylovorans*, and *Methanolobus* (Table S3). Among them, *Methanomethylovorans* and *Methanolobus* are methyltrophic methanogens, while *Methanothermobacter*, *Methanobacterium*, *Methanococcus, and Methanocalculus* are CO_2_-reducing methanogens.

Metabolic gene clone libraries and QPCR analysis demonstrate that NRB, SRB, and methanogens were ubiquitous in the reservoir. The NRB mainly belonged to *Pseudomonas*, *Azospirillum*, *Bradyrhizobium*, *Thauera*, *Magnetospirillum*, *Sinorhizobium*, *Azoarcus*, and *Rhodobacter*. The SRB were *Desulfarculus*, *Desulfomonile*, *Desulfosarcina*, *Desulfotignum*, *Desulfacinum*, *Desulfatibacillum*, *Desulfatibacillum*, *Desulfomicrobium*, and *Desulfovibrio*. As reported in previous research (Wang et al. 2012; Zhao et al. 2012), the majority of the archaea identified in the reservoir were methanogens, including methyltrophic, acetoclastic, and CO_2_-reducing *Methanomethylovorans*, *Methanosaeta*, *Methanococcus*, *Methanolobus*, and *Methanobacterium* (Liu and Whitman 2008).

It is noteworthy that oil reservoirs have low redox potential and therefore harbor abundant anaerobic and facultative microorganisms. However, abundant aerobic microorganisms were also detected in the production well, and anaerobic microorganisms were also detected in the injected water. These microbial populations included *Pseudomonas*, *Sphingomonas*, *Ochrobactrum*, *Dietzia*, *Arcobacter*, *Halomonas*, *Marinobacterium,* and methanogens. This phenomenon may be closely related to the microbial populations in the injected water passing through reservoir strata and reaching production wells, while the produced water was collected and injected into the reservoir.

In summary, 16S rRNA gene miseq sequencing, metabolic gene clone libraries, and QPCR analysis indicate that abundant microbial populations, including HDB, NRB, SRB, and methanogens, are ubiquitous in the Luliang water-flooding reservoir. These results also suggest that this reservoir has potential for MEOR and biological control of SRB propagation by stimulating NRB.

## Supporting Information

Figure S1 The number of bacterial and archaeal sequences obtained from the injection and production water samples by 16S rRNA miseq sequencing. **Figure S2.** The relative proportion of bacterial populations at phylum level in the injection and production water samples obtained from Luliang reservoir. **Table S1.** The 155 bacterial genera detected in Luliang reservoir by 16S rRNA miseq sequencing. **Table S2.** The bacterial populations with alkane monooxygenase gene (*alk*) obtained from RDP's FunGene. **Table S3.** The archaeal genera detected in Luliang reservoir by 16S rRNA miseq sequencing.
