# Supplementary material for: Microbial diversity and abundance in the Xinjiang Luliang long-term water-flooding petroleum reservoir
Source: Microbiologyopen. 2015 Feb 2;4(2):332–42. doi: 10.1002/mbo3.241 (PMC4398513; doi:10.1002/mbo3.241)
Supplement: Figure S1 — The number of bacterial and archaeal sequences obtained from the injection and production water samples by 16S rRNA miseq sequencing. Figure S2. The relative proportion of bacterial populations at phylum level in the injection and production water samples obtained from Luliang reservoir. Table S1. The 155 bacterial genera detected in Luliang reservoir by 16S rRNA miseq sequencing. Table S2. The bacterial populations with alkane monooxygenase gene (alk) obtained from RDP's FunGene. Table S3. The archaeal genera detected in Luliang reservoir by 16S rRNA miseq sequencing. [file mbo30004-0332-sd1.doc]

Supporting Information

**Microbial diversity and abundance in the Xinjiang Luliang long-term water-flooding petroleum reservoir**

Peike Gao^1, 2^, Huimei Tian^1, 2^, Guoqiang Li^1, 2^, Hongwen Sun^3^ & Ting Ma^1, 2^

^1^ Key Laboratory of Molecular Microbiology and Technology, Ministry of Education, Tianjin 300071, P.R. China

^2^ College of Life Sciences, Nankai University, Tianjin 300071, P.R. China

^3^ College of Environmental Science and Engineering, Nankai University, Tianjin 300071, P.R. China

**Correspondence**

Ting Ma, College of Life Sciences, Nankai University, Tianjin 300071, P.R. China. Tel/Fax: 86-22-23498185. E-mail: [tingma@nankai.edu.cn](mailto:tingma@nankai.edu.cn)

**Conflict of interest**

The authors declare that obviously there is no conflict of interest regarding the publication of this article.

**Supporting information**

Additional Supporting Information may be found in the attachment

**This document file includes:**

Supplementary Text

Supplementary Figure: Fig. S1 and S2

Supplementary Table: Table S1 to S3

***2.3 Clone library construction of napA, dsrB and mcrA genes***

***(1) The PCR reaction mixtures and PCR conditions for napA gene clone library***

PCR amplification of *napA* gene was conducted in 25 µl reactions with 4.5 µL sterile ddH_2_O, 5 µL 5× *TransStart®FastPfu* Buffer (TransGen Biotech, China), 2.5 µL 2.5 mM dNTPs, 1 µL *TransStart® FastPfu* DNA Polymerase, 5 µL forward primer (20 μM), 5 µL reverse primer (20 μM), and 2 µL genomic DNA (100 ng). The PCR amplification protocol for *napA* gene consisted of an initial denaturation at 95°C for 4 min, followed by 35 cycles at 94 °C for 1min, at 52 °C for 1 min, and at 72 °C for 1 min, with a final extension step at 72 °C for 10 min.

***(2) The PCR reaction mixtures and PCR conditions for dsrB gene clone library***

For *dsrB* gene, PCR was performed in a total volume 25 µl. Each PCR mixture contained 12.5 µL sterile ddH_2_O, 5 µL 5× *TransStart®FastPfu* Buffer (TransGen Biotech, China), 2.5 µL 2.5 mM dNTPs, 1 µL *TransStart® FastPfu* DNA Polymerase, 1 µL forward primer (20 μM), 1 µL reverse primer (20 μM), and 2 µL genomic DNA (100 ng). Thermal cycling was carried out by using an initial denaturation step of 94 °C for 4 min, followed by 35 cycles of denaturation at 94 °C for 1 min, annealing at 55 °C for 1 min, and elongation at 72 °C.

***(3) The PCR reaction mixtures and PCR conditions for mcrA gene clone library***

The PCRs were carried out in triplicate using 25 µl reactions with 12.5 µL sterile ddH_2_O, 5 µL 5× *TransStart®FastPfu* Buffer (TransGen Biotech, China), 2.5 µL 2.5 mM dNTPs, 1 µL *TransStart® FastPfu* DNA Polymerase, 1 µL forward primer (20 μM), 1 µL reverse primer (20 μM), and 2 µL genomic DNA (100 ng). Reaction conditions included an initial denaturation step at 95 °C for 3 min, followed by five cycles of denaturation at 95 °C for 30 s, annealing at 48 °C for 45 s, and extension at 72 °C for 30 s, with a ramp rate of 0.1 °C/s from the annealing to the extension temperature. These initial five cycles were followed with 30 cycles of denaturation at 95 °C for 30 s, annealing at 55 °C for 45 s, and extension at 72 °C for 30 s, followed by a final extension step at 72 °C for 10 min.

**Fig. S1** The number of bacterial and archaeal sequences obtained from the injection and production water samples by 16S rRNA miseq-sequencing.

**Fig. S2** The relative proportion of bacterial populations at phylum level in the injection and production water samples obtained from Luliang reservoir.

**Table S1** The 155 bacterial genera detected in Luliang reservoir by 16S rRNA miseq-sequencing

| Taxa (Phylum-Class-Order-Family-Genus) | Lu3084 | Lu3065 | Lu3064 | Lu3096 |
| --- | --- | --- | --- | --- |
| Actinobacteria; c_Actinobacteria; o_Actinomycetales; f_Corynebacteriaceae; g_Corynebacterium | 0.06309 | 0.08676 | 0.00000 | 0.01589 |
| Actinobacteria; c_Actinobacteria; o_Actinomycetales; f_Dermabacteraceae; g_Brachybacterium | 0.00000 | 0.03155 | 0.00000 | 0.00000 |
| Actinobacteria; c_Actinobacteria; o_Actinomycetales; f_Dietziaceae; g_Dietzia | 0.96215 | 0.78871 | 0.65665 | 0.66730 |
| Actinobacteria; c_Actinobacteria; o_Actinomycetales; f_Intrasporangiaceae; g_Phycicoccus | 0.00000 | 0.00789 | 0.00000 | 0.00000 |
| Actinobacteria; c_Actinobacteria; o_Actinomycetales; f_Microbacteriaceae; g_Microbacterium | 0.00789 | 0.01577 | 0.00000 | 0.02383 |
| Actinobacteria; c_Actinobacteria; o_Actinomycetales; f_Micrococcaceae; g_Kocuria | 0.00000 | 0.00789 | 0.00000 | 0.00000 |
| Actinobacteria; c_Actinobacteria; o_Actinomycetales; f_Micrococcaceae; g_Nesterenkonia | 0.00000 | 0.00789 | 0.00000 | 0.00000 |
| Actinobacteria; c_Actinobacteria; o_Actinomycetales; f_Mycobacteriaceae; g_Mycobacterium | 0.03943 | 0.03155 | 0.02373 | 0.00794 |
| Actinobacteria; c_Actinobacteria; o_Actinomycetales; f_Nocardiaceae; g_Rhodococcus | 0.32334 | 0.37069 | 0.18987 | 0.41309 |
| Actinobacteria; c_Actinobacteria; o_Actinomycetales; f_Nocardioidaceae; g_Aeromicrobium | 0.01577 | 0.00000 | 0.00000 | 0.00000 |
| Actinobacteria; c_Actinobacteria; o_Actinomycetales; f_Propionibacteriaceae; g_Propionibacterium | 0.13407 | 0.38647 | 0.00000 | 0.02383 |
| Actinobacteria; c_Actinobacteria; o_Actinomycetales; f_Tsukamurellaceae; g_Tsukamurella | 0.04732 | 0.00000 | 0.00000 | 0.00000 |
| Actinobacteria; c_Rubrobacteria; o_Rubrobacterales; f_Rubrobacteraceae; g_Rubrobacter | 0.00789 | 0.00000 | 0.00000 | 0.00000 |
| Actinobacteria; c_Thermoleophilia; o_Solirubrobacterales; f_Patulibacteraceae; g_Patulibacter | 0.01577 | 0.01577 | 0.00000 | 0.02383 |
| Bacteroidetes; c_Bacteroidia; o_Bacteroidales; f_Bacteroidaceae; g_Bacteroides | 0.12618 | 0.09464 | 0.14241 | 0.45281 |
| Bacteroidetes; c_Bacteroidia; o_Bacteroidales; f_Porphyromonadaceae; g_Parabacteroides | 0.00789 | 0.00789 | 0.00791 | 0.03972 |
| Bacteroidetes; c_Bacteroidia; o_Bacteroidales; f_Prevotellaceae; g_Prevotella | 0.01577 | 0.00000 | 0.00000 | 0.00794 |
| Bacteroidetes; c_Flavobacteriia; o_Flavobacteriales; f_Flavobacteriaceae; g_Aequorivita | 0.00789 | 0.00000 | 0.00000 | 0.00000 |
| Bacteroidetes; c_Flavobacteriia; o_Flavobacteriales; f_Flavobacteriaceae; g_Flavobacterium | 0.02366 | 0.11042 | 0.02373 | 0.03178 |
| Bacteroidetes; c_Flavobacteriia; o_Flavobacteriales; f_Flavobacteriaceae; g_Muricauda | 0.00000 | 0.00000 | 0.00791 | 0.00000 |
| Bacteroidetes; c_Sphingobacteriia; o_Sphingobacteriales; f_Amoebophilaceae; g_Candidatus Amoebophilus | 0.00000 | 0.00000 | 0.00000 | 0.00794 |
| Bacteroidetes; c_Sphingobacteriia; o_Sphingobacteriales; f_Flexibacteraceae; g_Hymenobacter | 0.00000 | 0.00000 | 0.00000 | 0.00794 |
| Chlamydiae; c_Chlamydiia; o_Chlamydiales; f_Parachlamydiaceae; g_Candidatus Protochlamydia | 0.00000 | 0.13408 | 0.00000 | 0.00000 |
| Chloroflexi; c_Anaerolineae; o_Anaerolineales; f_Anaerolinaceae; g_Bellilinea | 0.00000 | 0.00000 | 0.03956 | 0.00000 |
| Chloroflexi; c_Anaerolineae; o_Anaerolineales; f_Anaerolinaceae; g_C1_B004 | 0.05521 | 0.01577 | 0.30854 | 0.03178 |
| Chloroflexi; c_Anaerolineae; o_Anaerolineales; f_Anaerolinaceae; g_SHD-231 | 0.37066 | 0.03944 | 1.48734 | 0.03972 |
| Chloroflexi; c_Anaerolineae; o_Anaerolineales; f_Anaerolinaceae; g_T78 | 0.96215 | 0.11831 | 5.70411 | 0.11916 |
| Chloroflexi; c_Anaerolineae; o_Anaerolineales; f_Anaerolinaceae; g_WCHB1-05 | 0.01577 | 0.00000 | 0.08703 | 0.01589 |
| Cyanobacteria; c_Synechococcophycideae; o_Synechococcales; f_Synechococcaceae; g_Prochlorococcus | 0.00000 | 0.00000 | 0.02373 | 0.00000 |
| Deferribacteres; c_Deferribacteres; o_Deferribacterales; f_Deferribacteraceae; g_Flexistipes | 0.07098 | 0.11831 | 0.47468 | 0.02383 |
| Firmicutes; c_Bacilli; o_Bacillales; f_Bacillaceae; g_Bacillus | 0.07098 | 0.22084 | 0.09494 | 0.17477 |
| Firmicutes; c_Bacilli; o_Bacillales; f_Paenibacillaceae; g_Brevibacillus | 0.00789 | 0.00789 | 0.00000 | 0.00794 |
| Firmicutes; c_Bacilli; o_Bacillales; f_Paenibacillaceae; g_Paenibacillus | 0.06309 | 0.04732 | 0.07911 | 0.05561 |
| Firmicutes; c_Bacilli; o_Bacillales; f_Staphylococcaceae; g_Staphylococcus | 0.31546 | 0.00789 | 0.00791 | 0.00794 |
| Firmicutes; c_Bacilli; o_Exiguobacterales; f_Exiguobacteraceae; g_Exiguobacterium | 0.15773 | 0.14197 | 0.10285 | 0.15888 |
| Firmicutes; c_Bacilli; o_Lactobacillales; f_Carnobacteriaceae; g_Trichococcus | 0.22871 | 0.18140 | 0.14241 | 0.13505 |
| Firmicutes; c_Bacilli; o_Lactobacillales; f_Enterococcaceae; g_Enterococcus | 0.02366 | 0.05521 | 0.01582 | 0.00794 |
| Firmicutes; c_Bacilli; o_Lactobacillales; f_Lactobacillaceae; g_Lactobacillus | 0.11041 | 0.05521 | 0.00000 | 0.00000 |
| Firmicutes; c_Bacilli; o_Lactobacillales; f_Streptococcaceae; g_Lactococcus | 0.00000 | 0.00000 | 0.00791 | 0.00000 |
| Firmicutes; c_Clostridia; o_Clostridiales; f_Clostridiaceae; g_Clostridium | 0.31546 | 0.08676 | 0.12658 | 0.07944 |
| Firmicutes; c_Clostridia; o_Clostridiales; f_Clostridiaceae; g_Fusibacter | 0.07098 | 0.06310 | 0.08703 | 0.79441 |
| Firmicutes; c_Clostridia; o_Clostridiales; f_Clostridiaceae; g_Sedimentibacter | 0.00789 | 0.00000 | 0.00000 | 0.00000 |
| Firmicutes; c_Clostridia; o_Clostridiales; f_Clostridiaceae; g_Soehngenia | 0.00000 | 0.00789 | 0.01582 | 0.00000 |
| Firmicutes; c_Clostridia; o_Clostridiales; f_Eubacteriaceae; g_Acetobacterium | 0.07098 | 0.18929 | 0.02373 | 0.19860 |
| Firmicutes; c_Clostridia; o_Clostridiales; f_Peptococcaceae; g_Desulfitobacterium | 0.00000 | 0.00000 | 0.00000 | 0.00794 |
| Firmicutes; c_Clostridia; o_Clostridiales; f_Peptococcaceae; g_Desulfotomaculum | 0.00000 | 0.00000 | 0.00791 | 0.00000 |
| Firmicutes; c_Clostridia; o_Clostridiales; f_Peptococcaceae; g_Desulfurispora | 0.03943 | 0.00789 | 0.00791 | 0.00000 |
| Firmicutes; c_Clostridia; o_Clostridiales; f_Peptococcaceae; g_Niigata-25 | 0.00000 | 0.00000 | 0.00000 | 0.00794 |
| Firmicutes; c_Clostridia; o_Clostridiales; f_Peptococcaceae; g_Sporotomaculum | 0.00000 | 0.00000 | 0.00791 | 0.00000 |
| Firmicutes; c_Clostridia; o_Clostridiales; f_Ruminococcaceae; g_Anaerofilum | 0.01577 | 0.02366 | 0.03956 | 0.03972 |
| Firmicutes; c_Clostridia; o_Clostridiales; f_Veillonellaceae; g_vadinHB04 | 0.01577 | 0.00000 | 0.00791 | 0.00000 |
| Fusobacteria; c_Fusobacteria; o_Fusobacteriales; f_Leptotrichiaceae; g_Leptotrichia | 0.07886 | 0.00000 | 0.00000 | 0.00000 |
| Nitrospirae; c_Nitrospira; o_Nitrospirales; f_Nitrospiraceae; g_Nitrospira | 0.00000 | 0.03155 | 0.00000 | 0.00794 |
| Nitrospirae; c_Nitrospira; o_Nitrospirales; f_Thermodesulfovibrionaceae; g_DCE29 | 0.00000 | 0.07887 | 0.00791 | 0.00000 |
| Nitrospirae; c_Nitrospira; o_Nitrospirales; f_Thermodesulfovibrionaceae; g_Thermodesulfovibrio | 0.00000 | 0.00789 | 0.00000 | 0.00000 |
| Planctomycetes; c_Planctomycetia; o_Gemmatales; f_Gemmataceae; g_Gemmata | 0.00000 | 0.01577 | 0.00000 | 0.00000 |
| Proteobacteria; c_Alphaproteobacteria; o_Caulobacterales; f_Caulobacteraceae; g_Brevundimonas | 0.14984 | 0.50477 | 0.01582 | 0.07150 |
| Proteobacteria; c_Alphaproteobacteria; o_Caulobacterales; f_Caulobacteraceae; g_Caulobacter | 0.01577 | 0.11831 | 0.00000 | 0.00000 |
| Proteobacteria; c_Alphaproteobacteria; o_Caulobacterales; f_Caulobacteraceae; g_Mycoplana | 0.06309 | 0.11042 | 0.03956 | 0.08738 |
| Proteobacteria; c_Alphaproteobacteria; o_Caulobacterales; f_Caulobacteraceae; g_Phenylobacterium | 0.05521 | 0.00789 | 0.02373 | 0.00000 |
| Proteobacteria; c_Alphaproteobacteria; o_Kiloniellales; f_Kiloniellaceae; g_Thalassospira | 0.00000 | 0.02366 | 0.03165 | 0.02383 |
| Proteobacteria; c_Alphaproteobacteria; o_Rhizobiales; f_Bradyrhizobiaceae; g_Bradyrhizobium | 0.00789 | 0.00000 | 0.00000 | 0.00000 |
| Proteobacteria; c_Alphaproteobacteria; o_Rhizobiales; f_Brucellaceae; g_Ochrobactrum | 4.41640 | 5.46573 | 0.21361 | 0.21449 |
| Proteobacteria; c_Alphaproteobacteria; o_Rhizobiales; f_Hyphomicrobiaceae; g_Devosia | 0.08675 | 0.12619 | 0.02373 | 0.05561 |
| Proteobacteria; c_Alphaproteobacteria; o_Rhizobiales; f_Hyphomicrobiaceae; g_Parvibaculum | 0.33912 | 0.80448 | 0.48259 | 0.39720 |
| Proteobacteria; c_Alphaproteobacteria; o_Rhizobiales; f_Hyphomicrobiaceae; g_Rhodoplanes | 0.02366 | 0.00789 | 0.00000 | 0.00000 |
| Proteobacteria; c_Alphaproteobacteria; o_Rhizobiales; f_Methylobacteriaceae; g_Methylobacterium | 0.11830 | 0.13408 | 0.00000 | 0.00000 |
| Proteobacteria; c_Alphaproteobacteria; o_Rhizobiales; f_Phyllobacteriaceae; g_Nitratireductor | 0.00789 | 0.01577 | 0.00791 | 0.01589 |
| Proteobacteria; c_Alphaproteobacteria; o_Rhizobiales; f_Rhizobiaceae; g_Agrobacterium | 0.12618 | 0.14197 | 0.04747 | 0.16683 |
| Proteobacteria; c_Alphaproteobacteria; o_Rhizobiales; f_Xanthobacteraceae; g_Xanthobacter | 0.00789 | 0.00000 | 0.00000 | 0.00000 |
| Proteobacteria; c_Alphaproteobacteria; o_Rhodobacterales; f_Hyphomonadaceae; g_Hyphomonas | 0.53628 | 0.59942 | 0.98101 | 0.77852 |
| Proteobacteria; c_Alphaproteobacteria; o_Rhodobacterales; f_Hyphomonadaceae; g_Maricaulis | 0.37066 | 0.09464 | 0.35601 | 0.03972 |
| Proteobacteria; c_Alphaproteobacteria; o_Rhodobacterales; f_Hyphomonadaceae; g_Oceanicaulis | 0.71767 | 0.13408 | 0.44304 | 0.03178 |
| Proteobacteria; c_Alphaproteobacteria; o_Rhodobacterales; f_Rhodobacteraceae; g_Paracoccus | 0.00789 | 0.18140 | 0.00791 | 0.01589 |
| Proteobacteria; c_Alphaproteobacteria; o_Rhodobacterales; f_Rhodobacteraceae; g_Rhodobacter | 0.36278 | 0.04732 | 0.02373 | 0.03178 |
| Proteobacteria; c_Alphaproteobacteria; o_Rhodobacterales; f_Rhodobacteraceae; g_Rhodovulum | 0.10252 | 0.05521 | 0.04747 | 0.03972 |
| Proteobacteria; c_Alphaproteobacteria; o_Rhodobacterales; f_Rhodobacteraceae; g_Roseivivax | 0.00000 | 0.03155 | 0.00000 | 0.00000 |
| Proteobacteria; c_Alphaproteobacteria; o_Rhodospirillales; f_Acetobacteraceae; g_Roseomonas | 0.00000 | 0.02366 | 0.00000 | 0.01589 |
| Proteobacteria; c_Alphaproteobacteria; o_Rhodospirillales; f_Rhodospirillaceae; g_Azospirillum | 0.70978 | 0.56787 | 0.75158 | 0.09533 |
| Proteobacteria; c_Alphaproteobacteria; o_Rhodospirillales; f_Rhodospirillaceae; g_Magnetospirillum | 0.14984 | 0.03944 | 0.50633 | 0.01589 |
| Proteobacteria; c_Alphaproteobacteria; o_Rhodospirillales; f_Rhodospirillaceae; g_Novispirillum | 1.21451 | 0.41801 | 1.16297 | 0.18271 |
| Proteobacteria; c_Alphaproteobacteria; o_Rhodospirillales; f_Rhodospirillaceae; g_Oleomonas | 0.00000 | 0.00000 | 0.01582 | 0.00000 |
| Proteobacteria; c_Alphaproteobacteria; o_Rhodospirillales; f_Rhodospirillaceae; g_Phaeospirillum | 0.00789 | 0.02366 | 0.02373 | 0.00794 |
| Proteobacteria; c_Alphaproteobacteria; o_Rhodospirillales; f_Rhodospirillaceae; g_Rhodospirillum | 0.07098 | 0.00000 | 0.03165 | 0.00794 |
| Proteobacteria; c_Alphaproteobacteria; o_Rickettsiales; f_Rickettsiaceae; g_Wolbachia | 0.00789 | 0.00000 | 0.00000 | 0.00000 |
| Proteobacteria; c_Alphaproteobacteria; o_Sphingomonadales; f_Erythrobacteraceae; g_Erythrobacter | 0.00000 | 0.04732 | 0.07120 | 0.00794 |
| Proteobacteria; c_Alphaproteobacteria; o_Sphingomonadales; f_Erythrobacteraceae; g_Erythromicrobium | 0.00789 | 0.00789 | 0.00000 | 0.00000 |
| Proteobacteria; c_Alphaproteobacteria; o_Sphingomonadales; f_Sphingomonadaceae; g_Novosphingobium | 0.83596 | 0.55998 | 0.18987 | 0.03972 |
| Proteobacteria; c_Alphaproteobacteria; o_Sphingomonadales; f_Sphingomonadaceae; g_Sphingomonas | 2.52366 | 3.28102 | 0.27690 | 0.11916 |
| Proteobacteria; c_Alphaproteobacteria; o_Sphingomonadales; f_Sphingomonadaceae; g_Sphingopyxis | 0.05521 | 0.11831 | 0.05538 | 0.00794 |
| Proteobacteria; c_Betaproteobacteria; o_Burkholderiales; f_Alcaligenaceae; g_Achromobacter | 0.14196 | 0.05521 | 0.01582 | 0.01589 |
| Proteobacteria; c_Betaproteobacteria; o_Burkholderiales; f_Alcaligenaceae; g_Pigmentiphaga | 0.00000 | 0.03944 | 0.03165 | 0.00000 |
| Proteobacteria; c_Betaproteobacteria; o_Burkholderiales; f_Burkholderiaceae; g_Burkholderia | 0.00000 | 0.07887 | 0.00000 | 0.00000 |
| Proteobacteria; c_Betaproteobacteria; o_Burkholderiales; f_Comamonadaceae; g_Acidovorax | 0.44164 | 0.82025 | 0.05538 | 0.03178 |
| Proteobacteria; c_Betaproteobacteria; o_Burkholderiales; f_Comamonadaceae; g_Alicycliphilus | 0.00000 | 0.00000 | 0.00791 | 0.00000 |
| Proteobacteria; c_Betaproteobacteria; o_Burkholderiales; f_Comamonadaceae; g_Aquabacterium | 0.03943 | 0.18140 | 0.04747 | 0.01589 |
| Proteobacteria; c_Betaproteobacteria; o_Burkholderiales; f_Comamonadaceae; g_Hydrogenophaga | 0.03155 | 0.01577 | 0.01582 | 0.01589 |
| Proteobacteria; c_Betaproteobacteria; o_Burkholderiales; f_Comamonadaceae; g_Hylemonella | 0.48107 | 0.40224 | 1.96203 | 0.43692 |
| Proteobacteria; c_Betaproteobacteria; o_Burkholderiales; f_Comamonadaceae; g_Rubrivivax | 0.04732 | 0.05521 | 0.00000 | 0.00794 |
| Proteobacteria; c_Betaproteobacteria; o_Burkholderiales; f_Oxalobacteraceae; g_Polynucleobacter | 0.00000 | 0.03155 | 0.00000 | 0.00000 |
| Proteobacteria; c_Betaproteobacteria; o_Hydrogenophilales; f_Hydrogenophilaceae; g_Thiobacillus | 0.01577 | 0.03155 | 0.00791 | 0.00000 |
| Proteobacteria; c_Betaproteobacteria; o_Methylophilales; f_Methylophilaceae; g_Methylotenera | 0.02366 | 0.00000 | 0.00000 | 0.00000 |
| Proteobacteria; c_Betaproteobacteria; o_Procabacteriales; f_Procabacteriaceae; g_Procabacter | 0.00000 | 0.01577 | 0.00791 | 0.01589 |
| Proteobacteria; c_Betaproteobacteria; o_Rhodocyclales; f_Rhodocyclaceae; g_Azovibrio | 0.06309 | 0.06310 | 0.45886 | 0.05561 |
| Proteobacteria; c_Betaproteobacteria; o_Rhodocyclales; f_Rhodocyclaceae; g_Thauera | 0.41009 | 0.54421 | 0.75158 | 0.76263 |
| Proteobacteria; c_Deltaproteobacteria; o_Bdellovibrionales; f_Bacteriovoracaceae; g_Bacteriovorax | 0.01577 | 0.09464 | 0.19778 | 0.15888 |
| Proteobacteria; c_Deltaproteobacteria; o_Desulfobacterales; f_Desulfobulbaceae; g_Desulfobulbus | 0.01577 | 0.00000 | 0.03165 | 0.01589 |
| Proteobacteria; c_Deltaproteobacteria; o_Desulfovibrionales; f_Desulfomicrobiaceae; g_Desulfomicrobium | 0.00789 | 0.03155 | 0.03956 | 0.03178 |
| Proteobacteria; c_Deltaproteobacteria; o_Desulfovibrionales; f_Desulfovibrionaceae; g_Desulfovibrio | 0.08675 | 0.12619 | 0.08703 | 1.04862 |
| Proteobacteria; c_Deltaproteobacteria; o_Desulfuromonadales; f_Geobacteraceae; g_Geobacter | 0.00789 | 0.00000 | 0.00000 | 0.00000 |
| Proteobacteria; c_Deltaproteobacteria; o_Syntrophobacterales; f_Desulfobacteraceae; g_Desulfobacterium | 0.00000 | 0.00000 | 0.02373 | 0.00000 |
| Proteobacteria; c_Deltaproteobacteria; o_Syntrophobacterales; f_Desulfobacteraceae; g_Desulfotignum | 0.05521 | 0.00789 | 0.28481 | 0.02383 |
| Proteobacteria; c_Deltaproteobacteria; o_Syntrophobacterales; f_Syntrophobacteraceae; g_Syntrophobacter | 0.18139 | 0.00789 | 0.03956 | 0.00000 |
| Proteobacteria; c_Epsilonproteobacteria; o_Campylobacterales; f_Campylobacteraceae; g_Arcobacter | 1.70347 | 2.50020 | 1.82753 | 20.1621 |
| Proteobacteria; c_Epsilonproteobacteria; o_Campylobacterales; f_Campylobacteraceae; g_Sulfurospirillum | 0.00789 | 0.27605 | 0.02373 | 0.06355 |
| Proteobacteria; c_Epsilonproteobacteria; o_Campylobacterales; f_Helicobacteraceae; g_Sulfurimonas | 0.13407 | 0.18140 | 0.09494 | 0.36543 |
| Proteobacteria; c_Gammaproteobacteria; o_Alteromonadales; f_[Chromatiaceae]; g_Alishewanella | 0.00789 | 0.03944 | 0.00791 | 0.06355 |
| Proteobacteria; c_Gammaproteobacteria; o_Alteromonadales; f_Alteromonadaceae; g_Marinobacter | 0.40221 | 0.89124 | 0.50633 | 1.19956 |
| Proteobacteria; c_Gammaproteobacteria; o_Alteromonadales; f_Idiomarinaceae; g_Pseudidiomarina | 0.38644 | 0.78871 | 0.41930 | 0.57197 |
| Proteobacteria; c_Gammaproteobacteria; o_Alteromonadales; f_Shewanellaceae; g_Shewanella | 4.14826 | 4.79533 | 1.17089 | 2.08135 |
| Proteobacteria; c_Gammaproteobacteria; o_Chromatiales; f_Halothiobacillaceae; g_Halothiobacillus | 0.00000 | 0.00789 | 0.00000 | 0.00794 |
| Proteobacteria; c_Gammaproteobacteria; o_Enterobacteriales; f_Enterobacteriaceae; g_Trabulsiella | 0.66246 | 0.69406 | 0.40348 | 0.75469 |
| Proteobacteria; c_Gammaproteobacteria; o_Methylococcales; f_Methylococcaceae; g_Methylomonas | 0.00000 | 0.00000 | 0.02373 | 0.00000 |
| Proteobacteria; c_Gammaproteobacteria; o_Oceanospirillales; f_Alcanivoracaceae; g_Alcanivorax | 0.01577 | 0.01577 | 0.00791 | 0.00000 |
| Proteobacteria; c_Gammaproteobacteria; o_Oceanospirillales; f_Halomonadaceae; g_Candidatus Portiera | 0.00000 | 0.00000 | 0.00791 | 0.00000 |
| Proteobacteria; c_Gammaproteobacteria; o_Oceanospirillales; f_Halomonadaceae; g_Halomonas | 13.8880 | 10.6318 | 0.30854 | 0.34160 |
| Proteobacteria; c_Gammaproteobacteria; o_Oceanospirillales; f_Oceanospirillaceae; g_Marinobacterium | 2.42902 | 3.20215 | 6.68513 | 10.1366 |
| Proteobacteria; c_Gammaproteobacteria; o_Oceanospirillales; f_Oceanospirillaceae; g_Nitrincola | 0.00000 | 0.00789 | 0.00000 | 0.00794 |
| Proteobacteria; c_Gammaproteobacteria; o_Oceanospirillales; f_Oceanospirillaceae; g_Oleibacter | 0.44953 | 3.73058 | 2.39715 | 0.41309 |
| Proteobacteria; c_Gammaproteobacteria; o_Pseudomonadales; f_Moraxellaceae; g_Acinetobacter | 0.63880 | 1.07264 | 0.15823 | 0.25421 |
| Proteobacteria; c_Gammaproteobacteria; o_Pseudomonadales; f_Moraxellaceae; g_Enhydrobacter | 0.03943 | 0.07887 | 0.00791 | 0.00794 |
| Proteobacteria; c_Gammaproteobacteria; o_Pseudomonadales; f_Moraxellaceae; g_Psychrobacter | 0.00000 | 0.00789 | 0.00000 | 0.00000 |
| Proteobacteria; c_Gammaproteobacteria; o_Pseudomonadales; f_Pseudomonadaceae; g_Azorhizophilus | 0.00000 | 0.00789 | 0.00000 | 0.00000 |
| Proteobacteria; c_Gammaproteobacteria; o_Pseudomonadales; f_Pseudomonadaceae; g_Pseudomonas | 1.11199 | 3.52552 | 1.03639 | 5.47347 |
| Proteobacteria; c_Gammaproteobacteria; o_Thiotrichales; f_Piscirickettsiaceae; g_Methylophaga | 0.11830 | 0.14197 | 0.08703 | 1.23133 |
| Proteobacteria; c_Gammaproteobacteria; o_Vibrionales; f_Vibrionaceae; g_Vibrio | 0.00789 | 0.01577 | 0.00791 | 0.06355 |
| Proteobacteria; c_Gammaproteobacteria; o_Xanthomonadales; f_Xanthomonadaceae; g_Arenimonas | 0.00789 | 0.03155 | 0.03956 | 0.00794 |
| Proteobacteria; c_Gammaproteobacteria; o_Xanthomonadales; f_Xanthomonadaceae; g_Pseudoxanthomonas | 0.03155 | 0.41013 | 0.06329 | 0.03972 |
| Proteobacteria; c_Gammaproteobacteria; o_Xanthomonadales; f_Xanthomonadaceae; g_Rhodanobacter | 0.00789 | 0.00000 | 0.00000 | 0.00794 |
| Spirochaetes; c_Spirochaetes; o_Sphaerochaetales; f_Sphaerochaetaceae; g_Sphaerochaeta | 0.02366 | 0.03944 | 0.13449 | 0.65141 |
| Spirochaetes; c_Spirochaetes; o_Spirochaetales; f_Spirochaetaceae; g_Treponema | 0.00789 | 0.00000 | 0.00791 | 0.02383 |
| Spirochaetes; c_WWE1; o_[Cloacamonales]; f_[Cloacamonaceae]; g_W22 | 0.04732 | 0.00000 | 0.00000 | 0.00000 |
| Synergistetes; c_Synergistia; o_Synergistales; f_Aminiphilaceae; g_Aminiphilus | 0.12618 | 0.02366 | 0.03165 | 0.33365 |
| Synergistetes; c_Synergistia; o_Synergistales; f_Dethiosulfovibrionaceae; g_Aminobacterium | 0.00000 | 0.00000 | 0.00000 | 0.00794 |
| Synergistetes; c_Synergistia; o_Synergistales; f_Dethiosulfovibrionaceae; g_Dethiosulfovibrio | 0.00000 | 0.00000 | 0.01582 | 0.01589 |
| Synergistetes; c_Synergistia; o_Synergistales; f_Dethiosulfovibrionaceae; g_HA73 | 0.23659 | 0.02366 | 0.05538 | 0.20655 |
| Synergistetes; c_Synergistia; o_Synergistales; f_Synergistaceae; g_vadinCA02 | 0.00000 | 0.01577 | 0.01582 | 0.00000 |
| Synergistetes; c_Synergistia; o_Synergistales; f_Thermovirgaceae; g_Thermovirga | 0.00000 | 0.00000 | 0.00000 | 0.00794 |
| Tenericutes; c_Mollicutes; o_Acholeplasmatales; f_Acholeplasmataceae; g_Acholeplasma | 0.02366 | 0.01577 | 0.02373 | 0.11916 |
| Thermi; c_Deinococci; o_Thermales; f_Thermaceae; g_Thermus | 0.00789 | 0.00000 | 0.00000 | 0.00794 |
| Thermotogae; c_Thermotogae; o_Thermotogales; f_Thermotogaceae; g_Kosmotoga | 0.52050 | 0.04732 | 6.03639 | 0.02383 |
| Thermotogae; c_Thermotogae; o_Thermotogales; f_Thermotogaceae; g_Petrotoga | 0.00000 | 0.00000 | 0.00000 | 0.00794 |
| Verrucomicrobia; c_Opitutae; o_[Cerasicoccales]; f_[Cerasicoccaceae]; g_Cerasicoccus | 0.00789 | 0.00000 | 0.00000 | 0.00000 |
| Verrucomicrobia; c_Opitutae; o_Opitutales; f_Opitutaceae; g_Opitutus | 0.03155 | 0.01577 | 0.03165 | 0.01589 |
| Verrucomicrobia; c_Verrucomicrobiae; o_Verrucomicrobiales; f_Verrucomicrobiaceae; g_Luteolibacter | 0.00000 | 0.00000 | 0.00000 | 0.00794 |
| Others | 52.3265 | 46.7150 | 57.5949 | 46.4887 |

**Table S2** The bacterial populations with alkane monooxygenase gene (*alk*) obtained from RDP's FunGene^★^

| **Phylum** | **Class** | **Order** | **Family** | **Genus** | **Species** |
| --- | --- | --- | --- | --- | --- |
| Proteobacteria | α- | Kordiimonadales | Kordiimonadaceae | Kordiimonas | Kordiimonas gwangyangensis |
|  |  | Parvularculales | Parvularculaceae | Parvularcula | Parvularcula bermudensis HTCC2503 |
|  |  | Rhizobiales | Aurantimonadaceae | Martelella | Martelella sp. S6-10 |
|  |  |  | Bradyrhizobiaceae | Rhodopseudomonas | Rhodopseudomonas sp. |
|  |  |  | Brucellaceae | Ochrobactrum | Ochrobactrum sp. ITRH1 |
|  |  |  | Hyphomicrobiaceae | Rhodomicrobium | Rhodomicrobium vannielii |
|  |  |  | Phyllobacteriaceae | Hoeflea | Hoeflea phototrophica |
|  |  |  | Rhizobiaceae | Agrobacterium | Agrobacterium sp. MS189b |
|  |  | Rhodospirillales | Acetobacteraceae | Acidiphilium | Acidiphilium cryptum JF-5 |
|  |  |  |  |  | Acidiphilium sp. PM |
|  |  |  |  |  | Acidiphilium multivorum |
|  |  |  |  | Acidisphaera | Acidisphaera sp. C197 |
|  |  |  | Rhodospirillaceae | Rhodospirillum | Rhodospirillum centenum |
|  |  | Rhodobacterales | Rhodobacteraceae | Ahrensia | Ahrensia sp. R2A130 |
|  |  |  |  | Citreicella | Citreicella sp. 357 |
|  |  |  |  | Dinoroseobacter | Dinoroseobacter shibae DFL 12 |
|  |  |  |  | Jannaschia | Jannaschia sp. CCS1 |
|  |  |  |  | Oceanicaulis | Oceanicaulis alexandrii HTCC2633 |
|  |  |  |  | Paracoccus | Paracoccus denitrificans PD1222 |
|  |  |  |  |  | Paracoccus sp. 1-C-2 |
|  |  |  |  | Phaeobacter | Phaeobacter gallaeciensis BS107 |
|  |  |  |  | Pseudovibrio | Pseudovibrio sp. FO-BEG1 |
|  |  |  |  | Rhodobacter | Rhodobacter capsulatus SB 1003 |
|  |  |  |  |  | Rhodobacter sphaeroides ATCC 17025 |
|  |  |  |  | Roseobacter | Roseobacter sp. CCS2 |
|  |  |  |  | Ruegeria | Ruegeria sp. TM1040 |
|  |  |  |  | Sagittula | Sagittula stellata E-37 |
|  | β- | Burkholderiales | Alcaligenaceae | Advenella | Advenella mimigardefordensis |
|  |  |  |  | Alcaligenes | Alcaligenes eutrophus |
|  |  |  |  |  | Alcaligenes sp. II-C-7 |
|  |  |  | Burkholderiaceae | Ralstonia | Ralstonia pickettii 12D |
|  |  |  |  |  | Ralstonia sp. 5_7_47FAA |
|  |  |  |  | Limnobacter | Limnobacter sp. MED105 |
|  |  |  |  | Burkholderia | Burkholderia ambifaria AMMD |
|  |  |  |  |  | Burkholderia cenocepacia |
|  |  |  |  |  | Burkholderia cepacia |
|  |  |  |  |  | Burkholderia gladioli BSR3 |
|  |  |  |  |  | Burkholderia glumae BGR1 |
|  |  |  |  |  | Burkholderia mallei ATCC 23344 |
|  |  |  |  |  | Burkholderia multivorans ATCC 17616 |
|  |  |  |  |  | Burkholderia phytofirmans PsJN |
|  |  |  |  |  | Burkholderia pseudomallei 1026a |
|  |  |  |  |  | Burkholderia testosterone |
|  |  |  |  |  | Burkholderia thailandensis E264 |
|  |  |  |  |  | Burkholderia vietnamiensis G4 |
|  |  |  |  |  | Burkholderia xenovorans LB400 |
|  |  |  | Comamonadaceae | Polaromonas | Polaromonas sp. JS666 |
|  |  |  |  | Variovorax | Variovorax paradoxus |
|  |  |  |  | Comamonas | Comamonas acidovorans |
|  |  |  |  | Delftia | Delftia acidovorans |
|  |  | Hydrogenophilales | Hydrogenophilaceae | Petrobacter | Petrobacter succinatimandens |
|  | γ- | Aeromonadales | Aeromonadaceae | Aeromonas |  |
|  |  | Alteromonadales | Alteromonadaceae | Glaciecola | Glaciecola punicea DSM 14233 |
|  |  |  |  | Marinobacter | Marinobacter adhaerens HP15 |
|  |  |  |  |  | Marinobacter algicola DG893 |
|  |  |  |  |  | Marinobacter aquaeolei VT8 |
|  |  |  |  |  | Marinobacter hydrocarbonoclasticus |
|  |  |  |  |  | Marinobacter manganoxydans MnI7-9 |
|  |  |  |  |  | Marinobacter sp. ELB17 |
|  |  |  | Shewanellaceae | Shewanella | Shewanella sp. Nah4 |
|  |  | Enterobacteriales | Enterobacteriaceae | Serratia | Serratia marcescens |
|  |  |  |  | Pantoea | Pantoea sp. BTRH11 |
|  |  | Legionellales | Legionellaceae | Legionella | Legionella longbeachae D-4968 |
|  |  |  |  |  | Legionella pneumophila 2300/99 Alcoy |
|  |  | Oceanospirillales | Alcanivoraceae | Alcanivorax | Alcanivorax borkumensis |
|  |  |  |  |  | Alcanivorax dieselolei |
|  |  |  |  |  | Alcanivorax hongdengensis |
|  |  |  |  |  | Alcanivorax jadensis |
|  |  |  |  |  | Alcanivorax venustensis |
|  |  |  |  | Sphingomonas | Sphingomonas paucimobilis |
|  |  |  |  |  | Sphingomonas yanoikuyae |
|  |  |  | Oceanospirillaceae | Thalassolituus | Thalassolituus oleivorans |
|  |  |  | Hahellaceae | Hahella | Hahella chejuensis KCTC 2396 |
|  |  | Pseudomonadales | Pseudomonadaceae | Pseudomonas | Pseudomonas aeruginosa |
|  |  |  |  |  | Pseudomonas chlororaphis subsp. aureofaciens |
|  |  |  |  |  | Pseudomonas extremaustralis 14-3 |
|  |  |  |  |  | Pseudomonas fluorescens |
|  |  |  |  |  | Pseudomonas mendocina NK-01 |
|  |  |  |  |  | Pseudomonas pseudoalcaligenes |
|  |  |  |  |  | Pseudomonas oleovorans |
|  |  |  |  |  | Pseudomonas putida |
|  |  |  |  |  | Pseudomonas stutzeri |
|  |  |  |  |  | Pseudomonas synxantha BG33R |
|  |  |  | Moraxellaceae | Psychrobacter | Psychrobacter sp. PRwf-1 |
|  |  |  |  | Acinetobacter | Acinetobacter baumannii |
|  |  |  |  |  | Acinetobacter calcoaceticus |
|  |  |  |  |  | Acinetobacter sp. 16BJ |
|  |  |  |  |  | Acinetobacter haemolyticus |
|  |  |  |  |  | Acinetobacter oleivorans DR1 |
|  |  |  |  |  | Acinetobacter radioresistens |
|  |  |  |  |  | Acinetobacter junii |
|  |  |  |  |  | Ac*inetobacter lwoffii* |
|  |  |  |  |  | Acinetobacter venetianus |
|  |  | Salinisphaerales | Salinisphaeraceae | Salinisphaera | Salinisphaera shabanensis |
|  |  | Xanthomonadales | Xanthomonadaceae | Stenotrophomonas | Stenotrophomonas sp. MS192a |
|  |  | Xanthomonadales | Xanthomonadaceae | Pseudoxanthomonas | Pseudoxanthomonas spadix BD-a59 |
|  |  |  |  | Rhodanobacter | Rhodanobacter spathiphylli B39 |
|  |  |  | Sinobacteraceae | Hydrocarboniphaga | Hydrocarboniphaga effusa AP103 |
|  | δ- | Myxococcales | Nannocystacea | Plesiocystis | Plesiocystis pacifica SIR-1 |
|  |  | Bdellovibrionales | Bdellovibrionaceae | Bdellovibrio | Bdellovibrio bacteriovorus HD100 |
| Firmicutes | Bacilli | Lactobacillales | Streptococcaceae | Streptococcus | Streptococcus salivarius SK126 |
|  |  | Bacillales | Bacillaceae | Exiguobacterium | Exiguobacterium aurantiacum |
|  |  |  |  | Bacillus | Bacillus flexus |
|  |  |  |  |  | Bacillus licheniformis |
|  |  |  |  |  | Bacillus subtilis |
|  |  |  |  |  | Bacillus pumilus |
|  |  |  |  |  | Bacillus cereus |
|  |  |  |  |  | Bacillus sp. BTRH40 |
|  |  |  |  | Geobacillus | Geobacillus gargensis |
|  |  |  |  |  | Geobacillus subterraneus |
|  |  |  |  |  | Geobacillus thermocatenulatus |
|  |  |  |  |  | Geobacillus thermoleovorans |
|  |  |  |  |  | Geobacillus stearothermophilus |
|  |  |  |  |  | Geobacillus thermodenitrificans |
|  |  |  |  |  | Geobacillus toebii |
|  |  |  |  |  | Geobacillus uzenensis |
| Actinobacteria | Actinobacteria | Actinomycetales | Catenulisporaceae | Catenulispora | Catenulispora acidiphila DSM 44928 |
|  |  |  |  | Caulobacter | Caulobacter sp. K31 |
|  |  |  | Dietziaceae | Dietzia | Dietzia cinnamea P4 |
|  |  |  |  |  | Dietzia sp. H0 |
|  |  |  | Corynebacteriaceae | Corynebacterium | Corynebacterium jeikeium ATCC 43734 |
|  |  |  |  |  | Corynebacterium deparraffinicum |
|  |  |  |  |  | Corynebacterium thermophilum |
|  |  |  |  |  | Corynebacterium variabile |
|  |  |  | Nocardiaceae | Gordonia | Gordonia araii NBRC 100433 |
|  |  |  |  |  | Gordonia alkanivorans |
|  |  |  |  |  | Gordonia desulfuricans |
|  |  |  |  |  | Gordonia nitida |
|  |  |  |  |  | Gordonia bronchialis DSM 43247 |
|  |  |  |  |  | Gordonia effusa NBRC 100432 |
|  |  |  |  |  | Gordonia neofelifaecis NRRL B-59395 |
|  |  |  |  |  | Gordonia otitidis NBRC 100426 |
|  |  |  |  |  | Gordonia sp. 30A |
|  |  |  |  |  | Gordonia sputi NBRC 100414 |
|  |  |  |  |  | Gordonia terrae NBRC 100016 |
|  |  |  |  | Rhodococcus | Rhodococcus equi ATCC 33707 |
|  |  |  |  |  | Rhodococcus erythropolis |
|  |  |  |  |  | Rhodococcus fascians |
|  |  |  |  |  | Rhodococcus imtechensis RKJ300 |
|  |  |  |  |  | Rhodococcus jostii RHA1 |
|  |  |  |  |  | Rhodococcus opacus B4 |
|  |  |  |  |  | Rhodococcus pyridinivorans AK37 |
|  |  |  |  |  | Rhodococcus rhodochrous |
|  |  |  |  | Nocardia | Nocardia brasiliensis ATCC 700358 |
|  |  |  |  |  | Nocardia cyriacigeorgica GUH-2 |
|  |  |  |  |  | Nocardia sp. |
|  |  |  | Mycobacteriaceae | Mycobacterium | Mycobacterium abscessus 3A-0119-R |
|  |  |  |  |  | Mycobacterium austroafricanum |
|  |  |  |  |  | Mycobacterium avium 104 |
|  |  |  |  |  | Mycobacterium bovis BCG str. Mexico |
|  |  |  |  |  | Mycobacterium colombiense CECT 3035 |
|  |  |  |  |  | Mycobacterium fortuitum |
|  |  |  |  |  | Mycobacterium gilvum PYR-GCK |
|  |  |  |  |  | Mycobacterium marinum |
|  |  |  |  |  | Mycobacterium massiliense 1S-151-0930 |
|  |  |  |  |  | Mycobacterium neoaurum |
|  |  |  |  |  | Mycobacterium parascrofulaceum ATCC BAA-614 |
|  |  |  |  |  | Mycobacterium phlei RIVM601174 |
|  |  |  |  |  | Mycobacterium rhodesiae JS60 |
|  |  |  |  |  | Mycobacterium smegmatis str. MC2 155 |
|  |  |  |  |  | Mycobacterium sp. JDM601 |
|  |  |  |  |  | Mycobacterium thermoresistibile ATCC 19527 |
|  |  |  |  |  | Mycobacterium tusciae JS617 |
|  |  |  |  |  | Mycobacterium ulcerans Agy99 |
|  |  |  |  |  | Mycobacterium vanbaalenii PYR-1 |
|  |  |  |  |  | Mycobacterium xenopi RIVM700367 |
|  |  |  |  | Amycolicicoccus | Amycolicicoccus subflavus DQS3-9A1 |
|  |  |  | Tsukamurellaceae | Tsukamurella | Tsukamurella paurometabola DSM 20162 |
|  |  |  | Segniliparaceae | Segniliparus | Segniliparus rotundus DSM 44985 |
|  |  |  | Frankiaceae | Frankia | Frankia alni ACN14a |
|  |  |  | Dermabacteraceae | Brachybacterium | Brachybacterium paraconglomeratum |
|  |  |  | Microbacteriaceae | Leucobacter | Leucobacter sp. 5-C-1 |
|  |  |  |  | Leifsonia | Leifsonia sp. S4-9 |
|  |  |  |  | Microbacterium | Microbacterium esteraromaticum |
|  |  |  |  |  | Microbacterium oxydans |
|  |  |  |  |  | Microbacterium schleiferi |
|  |  |  |  |  | Microbacterium sp. ITRH47 |
|  |  |  | Micrococcaceae | Arthrobacter | Arthrobacter sp. ITRH48 |
|  |  |  | Micromonosporaceae | Micromonospora | Micromonospora aurantiaca ATCC 27029 |
|  |  |  |  |  | Micromonospora lupini str. Lupac 08 |
|  |  |  |  |  | Micromonospora sp. L5 |
|  |  |  | Nocardioidaceae | Nocardioides | Nocardioides sp. CF8 |
|  |  |  |  |  | Nocardioides sp. JS614 |
|  |  |  |  |  | Nocardioides sp. MS274e |
|  |  |  |  | Aeromicrobium | Aeromicrobium marinum DSM 15272 |
|  |  |  | Pseudonocardiaceae | Amycolatopsis | Amycolatopsis mediterranei S699 |
|  |  |  |  | Saccharomonospora | Saccharomonospora paurometabolica YIM 90007 |
|  |  |  |  | Prauserella | Prauserella rugosa |
|  |  |  | Thermomonosporaceae | Thermomonospora | Thermomonospora curvata DSM 43183 |
|  |  | Rubrobacterales | Patulibacteraceae | Patulibacter | Patulibacter sp. I11 |
|  |  |  | Conexibacteraceae | Conexibacter | Conexibacter woesei DSM 14684 |
| Bacteroidetes | Sphingobacteria | Sphingobacteriales | Saprospiraceae | Haliscomenobacter | Haliscomenobacter hydrossis DSM 1100 |
|  |  |  | Flexibacteraceae | Microscilla | Microscilla marina ATCC 23134 |
|  |  |  |  | Runella | Runella slithyformis DSM 19594 |
|  |  |  | Sphingobacteriaceae | Pedobacter | Pedobacter sp. MS245e |
|  |  |  | Chitinophagaceae |  | Chitinophagaceae bacterium MS98c |
|  | Cytophagia | Cytophagales | Cytophagaceae | Fibrisoma | Fibrisoma limi BUZ 3 |
|  |  |  | Flammeovirgaceae | Marivirga | Marivirga tractuosa DSM 4126 |
|  |  |  | Cyclobacteriaceae | Nitritalea | Nitritalea halalk Baliphila LW7 |
|  | Flavobacteria | Flavobacteriales | Flavobacteriacea | Gaetbulibacter | Gaetbulibacter sp. S1-5 |
|  |  |  |  | Maribacter | Maribacter sp. HTCC2170 |
|  |  |  |  | Kordia | Kordia algicida OT-1 |
| Spirochaetes | Spirochaetes | Spirochaetales | Leptospiraceae | Leptospira | Leptospira licerasiae serovar Varillal str. VAR 010 |
|  |  |  |  | Turneriella | Turneriella parva DSM 21527 |

★ Based on RDP's FunGene (<http://fungene.cme.msu.edu//index.spr>), we gathered and categorized the bacterial populations with alkane monooxygenase gene (*alk*).

**Table S3 The archaeal genera detected in Luliang reservoir by 16S rRNA miseq-sequencing**

| Taxon (Phylum-Class-Order-Family-Genus) | Lu3064 | Lu3096 |
| --- | --- | --- |
| Crenarchaeota; c__Thaumarchaeota; o__Cenarchaeales; f__Cenarchaeaceae; g__Nitrosopumilus | 22.00062 | 0 |
| Euryarchaeota; c__Methanobacteria; o__Methanobacteriales; f__Methanobacteriaceae; g__Methanobacterium | 2.000056 | 0.980371 |
| Euryarchaeota; c__Methanobacteria; o__Methanobacteriales; f__Methanobacteriaceae; g__Methanothermobacter | 4.000112 | 0 |
| Euryarchaeota; c__Methanococci; o__Methanococcales; f__Methanococcaceae; g__Methanococcus | 0 | 1.960742 |
| Euryarchaeota; c__Methanomicrobia; o__Methanomicrobiales; f__Methanocorpusculaceae; g__Methanocalculus | 0 | 5.882225 |
| Euryarchaeota; c__Methanomicrobia; o__Methanosarcinales; f__Methanosarcinaceae; g__Methanolobus | 2.000056 | 87.253 |
| Euryarchaeota; c__Methanomicrobia; o__Methanosarcinales; f__Methanosarcinaceae; g__Methanomethylovorans | 20.00056 | 0.980371 |
| Others | 49.9986 | 2.943 |
